# Comparison of the diagnostic performance of machine learning algorithms for differentiating iron deficiency anemia and thalassemia

**DOI:** 10.1007/s00277-026-06894-5

**Published:** 2026-03-04

**Authors:** Yun Wang, Xu Yuan, Weiwei Xiao, Yanjun Lu

**Affiliations:** 1 Department of Laboratory Medicine, Tongji Hospital, Tongji Medical College, Huazhong University of Science and Technology, Wuhan, China; 2 Department of Laboratorial Medicine, Shenzhen Children’s Hospital, Shenzhen, 518038 Guangdong Province China

**Keywords:** Machine learning, Light gradient boosting, Iron deficiency anemia, Thalassemia

## Abstract

Accurate discrimination between iron deficiency anemia (IDA) and thalassemia trait (TT) is clinically essential for the effective management of patients with hypochromic microcytic anemia. Although numerous discrimination indices based on red blood cell (RBC) parameters have been proposed, their diagnostic accuracy remains suboptimal and highly population-specific. This study aimed to develop and validate a machine learning model to enhance discriminative performance. We utilized a derivation cohort of 376 patients (IDA, n = 186; TT, n = 190) for model development and internal validation, and a separate validation cohort of 196 patients for external testing. Five machine learning algorithms—Extreme Gradient Boosting (XGBoost), Logistic Regression (LR), Light Gradient Boosting Machine (LightGBM), Random Forest (RF), and AdaBoost—were trained and evaluated. Model performance was assessed using the area under the receiver operating characteristic curve (AUC), accuracy, sensitivity, specificity, positive predictive value (PPV), and negative predictive value (NPV). The LightGBM classifier achieved an AUC of 0.953 (accuracy 86.9%) in the internal validation set and 0.980 (accuracy 93.1%) in testing set. In the external validation cohort, the model demonstrated robust generalizability, attaining an AUC of 0.992 with an accuracy of 98.5%, sensitivity of 96.7%. Feature importance analysis identified mean corpuscular hemoglobin concentration (MCHC) and red cell distribution width-standard deviation (RDW-SD)mean as the most discriminative predictors. We developed and externally validated a robust LightGBM-based classifier that accurately discriminates between IDA and TT, offering clinicians with with a reliable, non-invasive decision-support tool for the differential diagnosis of microcytic anemia.

**Supplementary Information:**

The online version contains supplementary material available at 10.1007/s00277-026-06894-5.

## Introduction

Anemia is a common hematological disorder characterized by reduced red blood cell (RBC) count or hemoglobin concentration below thresholds defined by the World Health Organization (WHO): <12 g/dL in non-pregnant women, < 11 g/dL in pregnant women, and < 13 g/dL in men [1]. Among its various etiologies, iron deficiency anemia (IDA) and thalassemia trait (TT) are the two leading causes of hypochromic microcytic anemia globally [2–4]. IDA results from depleted iron stores due to inadequate dietary intake, chronic blood loss, or impaired absorption, accounting for approximately 66% of all anemia cases and affecting nearly one-quarter of the global population [5]. In contrast, thalassemia is an inherited hemoglobinopathy caused by mutations or deletions in the α- or β-globin genes. Approximately 7% of the global population are carriers of thalassemia variants, with an estimated 300,000 to 500,000 affected infants born annually. In China, the highest carrier rates are observed in southern provinces such as Guangdong, Guangxi, and Fujian [6].

Although IDA is both preventable and treatable with iron supplementation, thalassemia does not respond to iron therapy, and unnecessary supplementation in TT carriers may lead to iron overload with severe complications including hepatic, cardiac, and endocrine dysfunction [7]. Therefore, accurate and timely differentiation between IDA and TT is essential for guiding clinical management, genetic counseling, and prenatal screening programs.

The definitive diagnosis of IDA relies on biochemical markers of iron metabolism such as serum iron, transferrin saturation, ferritin, and total iron-binding capacity, whereas thalassemia requires hemoglobin electrophoresis, high-performance liquid chromatography (HPLC), or molecular genetic testing to identify globin gene mutations [8]. However, these confirmatory tests are expensive, time-consuming, and not universally available in resource-limited or high-throughput outpatient settings. As a result, numerous discriminant formulas using routine red blood cell indices—such as Mentzer, Green and King, England and Fraser, and Red Cell Distribution Width Index (RDWI)—have been developed to screen for and differentiate between IDA and TT [9, 10]. Nevertheless, the diagnostic performance of these indices varies significantly across populations. Factors including patient age, gender, ethnic background, and the specific genetic spectrum of thalassemia mutations can substantially influence their accuracy and utility [11]. Consequently, these formulas are best suited for preliminary screening rather than definitive diagnosis.

In recent years, machine learning (ML), a branch of artificial intelligence, has demonstrated considerable potential in identifying complex patterns within large datasets, enabling autonomous learning and accurate prediction. By leveraging medical data, ML techniques can significantly enhance diagnostic efficiency and support clinical decision-making. Several studies have already applied ML approaches to the differential diagnosis of IDA and TT, demonstrating superior performance compared to traditional discrimination indices [12–15]. In this study, we aimed to develop and validate a robust ML model for distinguishing TT from IDA using readily available laboratory data, thereby supporting clinical decision-making and reducing reliance on expensive confirmatory testing.

## Materials and methods

### Study design and population

This retrospective multicenter study utilized clinical data from three branches of Tongji Hospital in Wuhan, China: the Qiaokou Branch (Hankou District), the Optics Valley Branch (Wuhan East Lake High-Tech Development Zone), and the Sino-French New City Branch (Caidian District). Between January 2019 and May 2023, a total of 376 patients were enrolled for model development and internal validation, comprising 186 individuals with iron deficiency anemia (IDA) and 190 with thalassemia trait (TT). An additional independent cohort of 196 patients from the Optics Valley and Sino-French New City Branches was utilized for external validation.

The diagnosis of IDA was established based on iron metabolism criteria: serum iron < 30 ng/mL or transferrin saturation < 16% [16]. Thalassemia was confirmed using flow cytometry-based microarray hybridization, which detects 17 common β-thalassemia variants in the Chinese population, along with three large deletion α-globin gene defects (-SEA, -α3.7, and -α4.2), and three non-deletional α-thalassemia mutations (Hb Constant Spring [CS], Hb Quong Sze [QS], Hb Westmead [WS]). All thalassemia cases included in this study were phenotypically classified as thalassemia trait(mild microcytic anemia).

Patients were excluded if they met any of the following conditions: current iron supplementation therapy; concurrent IDA and TT; thyroid dysfunction; elevated vitamin B12 or folate levels; megaloblastic anemia; or systemic inflammatory disorders. The study was conducted in accordance with the Declaration of Helsinki and was approved by the Ethics Committee of Tongji Hospital (TJ-JRB2021S138). Owing to the retrospective design, the requirement for written informed consent was waived.

### Laboratory analysis process

Complete blood count analysis was performed using the Sysmex XN-9000 hematology analyzer (Sysmex Corporation, Japan). Serum iron and total iron‐binding capacity (TIBC) levels were measured by photometric assay on the Roche Cobas 8000 analyzer (Roche Diagnostics, Germany). Serum ferritin was determined by electrochemiluminescence immunoassay on the Cobas e 701 analyzer (Roche Diagnostics, Germany).

Genomic DNA was extracted from peripheral blood leukocytes using the Tianlong Magnetic Bead Nucleic Acid Extraction Kit (Tianlong, Xi’an, China). DNA concentration and purity were assessed with a NanoDrop ASP-2680 spectrophotometer (ACTGene, USA). Genetic testing for TT was performed by PCR-based flow fluorescence hybridization (Daan Gene, Guangzhou, China), designed to detect common Chinese mutations, including 17 β-thalassemia variants, three large deletion α-globin gene defects (-SEA, -α3.7, -α4.2), and three non-deletional α-globin mutations (Hb CS, Hb QS, Hb WS).

Genetic variants analyzed:

- *α-*Thalassemia:
  Deletions: -SEA (NC_000016.9:g.215400_234700del), -α3.7 (NC_000016.9:g.223300_227103del), ‐α4.2 (NC_000016.9:g.219817_223755del).
  Point Mutations: Hb CS (HBA2: c.427T > C), Hb QS (HBA2: c.377T > C), Hb WS (HBA2: c.427T > C).
- *β-*Thalassemia:
  A total of 17 mutations were screened, including: CD41-42 (‐CTTT) (HBB: c.126_129delCTTT), IVS‐II‐654 (C > T) (HBB: c.316–197 C > T), CD17 (A > T) (HBB: c.52 A > T), ‐28 (A > G) (HBB: c.‐78 A > G), CD26 (G > A) (HBB: c.79G > A; p.Glu26Lys), CD71‐72 (+ A) (HBB: c.216_217insA), CD43 (G > T) (HBB: c.130G > T), ‐29 (A > G) (HBB: c.‐79 A > G), Initiation Codon (ATG > AGG) (HBB: c.2T > G), CD14‐15 (+ G) (HBB: c.45_46insG), CD27‐28 (+ C) (HBB: c.84_85insC), ‐32 (C > A) (HBB: c.‐82 C > A), ‐30 (T > C) (HBB: c.‐80T > C), IVS‐I‐1 (G > T) (HBB: c.92 + 1G > T), IVS‐I‐5 (G > C) (HBB: c.92 + 5G > C), CD31 (‐C) (HBB: c.94delC), and Cap + 40‐43 (‐AAAC) (HBB: c.‐10_‐7delAAAC).

### Model fitting and evaluation

Feature selection was based on variables demonstrating statistically significant between-group differences, supplemented by predictors established in the literature. To mitigate multicollinearity, a correlation analysis was performed to exclude highly correlated variables. Optimal predictive features were subsequently identified through univariate analysis followed by multiple logistic regression.

For model development, Cohort 1 was randomly split into a training set (85%) and a test set (15%). A ten-fold cross-validation approach was employed, where the training data were partitioned into ten folds; each iteration utilized nine folds for training and one for validation. This process was repeated across all folds to improve model robustness and reduce the risk of overfitting.

Five machine learning algorithms were evaluated for their ability to distinguish thalassemia from IDA: Extreme Gradient Boosting (XGBoost), Logistic Regression (LR), Light Gradient Boosting Machine (LightGBM), Random Forest (RF), and AdaBoost. The model demonstrating superior performance during internal validation was selected for further analysis. An independent external validation was subsequently performed using Cohort 2. All predictive models were constructed and deployed on the Beckman Coulter DxAI platform (https://www.xsmartanalysis.com/beckman/login/). Model performance was assessed using the area under the ROC curve (AUC), accuracy, sensitivity, specificity, positive predictive value (PPV), and negative predictive value (NPV). Among the five algorithms tested, the LightGBM model achieved the highest overall discriminative performance. LightGBM is a gradient boosting framework that uses a leaf-wise tree growth strategy, offering rapid training, efficient memory use, and strong predictive capability.

### Statistical analysis

Continuous variables were presented as mean ± standard deviation (SD) or median (interquartile range (IQR)), depending on data distribution.Between-group comparisons were performed using the Mann-Whitney U test or one-way ANOVA, as appropriate. Categorical variables were analyzed using the Chi-square test or Fisher’s exact test, depending on the sample size and expected cell frequencies. ROC curve analysis was performed to identify the optimal cutoff values for parameters, maximizing both sensitivity and specificity. Statistical analyses were performed using SPSS version 22.0 (SPSS, Chicago, IL, USA). Statistical significance was determined as *P* < 0.05.

## Results

### Demographic characteristics of patients

A total of 572 patients were included in this study, comprising 291 with IDA and 281 with TT across two independent cohorts (Table 1). In cohort 1, the TT group had a significantly lower median age (9.5 years; range: 4.8–31.0) compared to the IDA group (13.0 years; range: 1.0–30.0; *p* < 0.05). Hemoglobin levels were significantly higher in the TT group, whereas mean corpuscular hemoglobin (MCH) was comparable between groups. Analysis of erythrocyte parameters revealed that the thalassemia group had significantly higher red blood cell (RBC) counts, hematocrit (HCT) and mean corpuscular hemoglobin concentration (MCHC), but lower mean corpuscular volume (MCV) and red cell distribution width (RDW) compared to the IDA group (all *p* < 0.05). Regarding leukocyte and platelet indices, the thalassemia group demonstrated higher neutrophil counts and percentages, while the IDA group showed significantly elevated platelet counts and plateletcrit (PCT). Iron metabolism profiles differed substantially between groups: serum iron, ferritin, and transferrin saturation (TSAT) were significantly reduced in IDA patients, whereas total iron-binding capacity (TIBC) and unsaturated iron-binding capacity (UIBC) were markedly increased compared to the thalassemia group (all *p* < 0.05). Similar results were also observed in Cohort 2.

**Table 1:** Baseline laboratory characteristics

	Cohort 1			Cohort 2		
	IDA (*n* = 186)	Thal (*n* = 190)	*p* value	IDA (*n* = 105)	Thal (*n* = 91)	*p* value
Sex						
Femal, n (%)	121 (65.05%)	103 (54.21%)	0.042	69 (65.71%)	45 (49.45%)	0.031
Male, n (%)	65 (34.95%)	87 (45.79%)		36 (34.29%)	46 (50.55%)	
Age	13.00 (1.02, 30.00)	9.50 (4.80, 31.00)	0.028	12.00 (1.10, 30.00)	10.00 (4.75, 29.50)	0.035
Hemoglobin (g/L)	93.11 (17.83)	107.30 (16.27)	< 0.001	92.72 (15.31)	107.94 (13.03)	< 0.001
MCH (pg)	20.05 (17.77, 23.27)	19.60 (18.32, 21.10)	0.444	20.00 (17.70, 23.10)	19.20 (18.50, 20.90)	0.729
MCHC (g/L)	294.00 (278.00, 309.00)	313.00 (306.25, 320.00)	< 0.001	297.00 (282.00, 311.00)	316.00 (309.00, 322.50)	< 0.001
RBC (×10^12/L)	4.55 (4.16, 5.00)	5.37 (4.98, 5.70)	< 0.001	4.50 (0.60)	5.35 (0.64)	< 0.001
MCV (fL)	68.60 (63.10, 76.08)	62.90 (59.12, 67.38)	< 0.001	67.90 (62.10, 74.80)	61.80 (59.20, 65.40)	< 0.001
RDW-CV (%)	17.70 (16.10, 20.10)	16.00 (15.10, 17.50)	< 0.001	18.04 (2.92)	16.71 (2.25)	< 0.001
RDW-SD (fL)	43.80 (41.02, 46.65)	36.00 (33.20, 38.80)	< 0.001	42.30 (39.50, 45.60)	34.50 (32.85, 37.50)	< 0.001
HCT (%)	31.40 (28.90, 34.20)	34.00 (31.52, 36.88)	< 0.001	30.97 (3.73)	34.08 (3.80)	< 0.001
sTfR(mg/L)	9.99 (6.46, 13.73)	4.60 (3.69, 5.68)	< 0.001	8.74 (6.07, 12.09)	4.72 (3.82, 5.52)	< 0.001
TIBC (µmol/L)	81.53 (74.28, 87.53)	55.13 (49.85, 61.02)	< 0.001	76.75 (69.28, 85.85)	54.54 (48.73, 59.49)	< 0.001
Serum iron (µmol/L)	4.06 (3.10, 5.73)	16.70 (12.58, 20.33)	< 0.001	4.10 (3.11, 6.15)	18.13 (13.80, 21.55)	< 0.001
Ferritin (ng/mL)	7.75 (5.00, 13.57)	92.45 (57.83, 157.75)	< 0.001	7.00 (5.00, 10.40)	75.70 (44.55, 158.45)	< 0.001
TSAT (%)	5.00 (3.83, 7.75)	30.60 (21.68, 39.62)	< 0.001	5.10 (4.10, 8.05)	32.10 (25.18, 41.20)	< 0.001
Transferrin (g/L)	3.62 (3.28, 3.89)	2.43 (2.19, 2.75)	< 0.001	3.56 (0.52)	2.51 (0.48)	< 0.001
UIBC (µmol/L)	75.40 (69.05, 82.50)	38.60 (30.40, 46.02)	< 0.001	71.40 (65.10, 80.90)	36.20 (29.15, 42.70)	< 0.001
WBC (×10^9/L)	7.30 (5.47, 9.22)	7.34 (5.83, 9.09)	0.909	7.32 (5.39, 9.20)	7.13 (5.96, 8.59)	0.769
Neutrophil counts (×10^9/L)	2.96 (2.12, 4.36)	3.66 (2.76, 5.05)	0.001	2.76 (1.96, 3.87)	3.46 (2.56, 4.75)	0.002
Neutrophil (%)	53.05 (27.02, 65.75)	55.30 (43.58, 67.55)	0.029	49.20 (23.30, 58.30)	53.40 (39.90, 65.10)	0.020
Monocytes(×10^9/L)	0.49 (0.35, 0.63)	0.46 (0.35, 0.58)	0.134	2.34 (1.73, 5.02)	2.46 (1.78, 3.52)	0.436
Monocytes (%)	6.35 (5.30, 8.30)	6.20 (4.92, 7.40)	0.031	40.30 (29.90, 61.80)	36.50 (24.75, 49.70)	0.042
Lymphocytes(×10^9/L)	2.18 (1.59, 4.87)	2.33 (1.70, 3.42)	0.537	0.50 (0.40, 0.68)	0.48 (0.39, 0.56)	0.204
Lymphocytes (%)	36.95 (25.40, 61.60)	34.55 (25.02, 44.95)	0.065	7.10 (5.80, 8.60)	6.20 (5.55, 7.65)	0.022
Basophils(×10^9/L)	0.02 (0.01, 0.04)	0.02 (0.01, 0.04)	0.503	0.02 (0.01, 0.03)	0.02 (0.02, 0.04)	0.359
Basophils (%)	0.30 (0.20, 0.50)	0.30 (0.20, 0.50)	0.57	0.30 (0.20, 0.50)	0.30 (0.20, 0.50)	0.455
Eosinophils(×10^9/L)	0.13 (0.06, 0.24)	0.14 (0.07, 0.24)	0.761	0.12 (0.07, 0.23)	0.15 (0.07, 0.23)	0.638
Eosinophils (%)	1.90 (0.70, 3.48)	2.00 (1.00, 3.10)	0.832	1.90 (1.00, 3.40)	2.00 (1.00, 3.65)	0.848
Platelet count(×10^9/L)	345.00 (259.25, 478.00)	300.00 (240.25, 372.75)	< 0.001	338.00 (275.00, 432.00)	305.00 (256.50, 386.50)	0.09
Plateletcrit (%)	0.34 (0.27, 0.45)	0.33 (0.26, 0.38)	0.023	0.35 (0.27, 0.45)	0.32 (0.27, 0.39)	0.302
PDW (fL)	13.00 (11.50, 15.50)	13.50 (11.00, 15.60)	0.903	11.40 (10.35, 13.05)	11.60 (9.90, 13.00)	0.924
Large platelet ratio (%)	26.40 (21.40, 30.30)	28.10 (21.60, 32.60)	0.144	26.60 (22.55, 33.20)	26.15 (21.15, 31.25)	0.373

Data are presented as number (%), median (25th–75th percentile) or mean ± SD. Values in bold represent statistically significant differences (*p* < 0.05). *IDA* iron deficiency anemia, *Thal* Thalassemia, *MCH* mean corpuscular hemoglobin, *MCHC* mean corpuscular hemoglobin concentration, *RBC* red blood cell count, *MCV* mean corpuscular volume, *RDW-CV* red cell distribution width–coefficient of variation, *RDW-SD* red cell distribution width–standard deviation, *HCT* hematocrit, *sTfR* soluble transferrin receptor, *TIBC* total iron-binding capacity, *TSAT* transferrin saturation, *UIBC* unsaturated iron-binding capacity, *WBC* white blood cell count, *PDW* platelet distribution width, *P-LCR*large platelet ratio

## Establishment of ML model

### Model performance and feature importance

Following the feature selection protocol detailed in the Methods section, seven laboratory parameters were identified as the optimal discriminative features for distinguishing TT from IDA: MCHC, MCV, red cell distribution width-standard deviation (RDW-SD), red cell distribution width-coefficient of variation (RDW-CV), RBC, hemoglobin (Hb), and HCT.

**Fig. 1:**
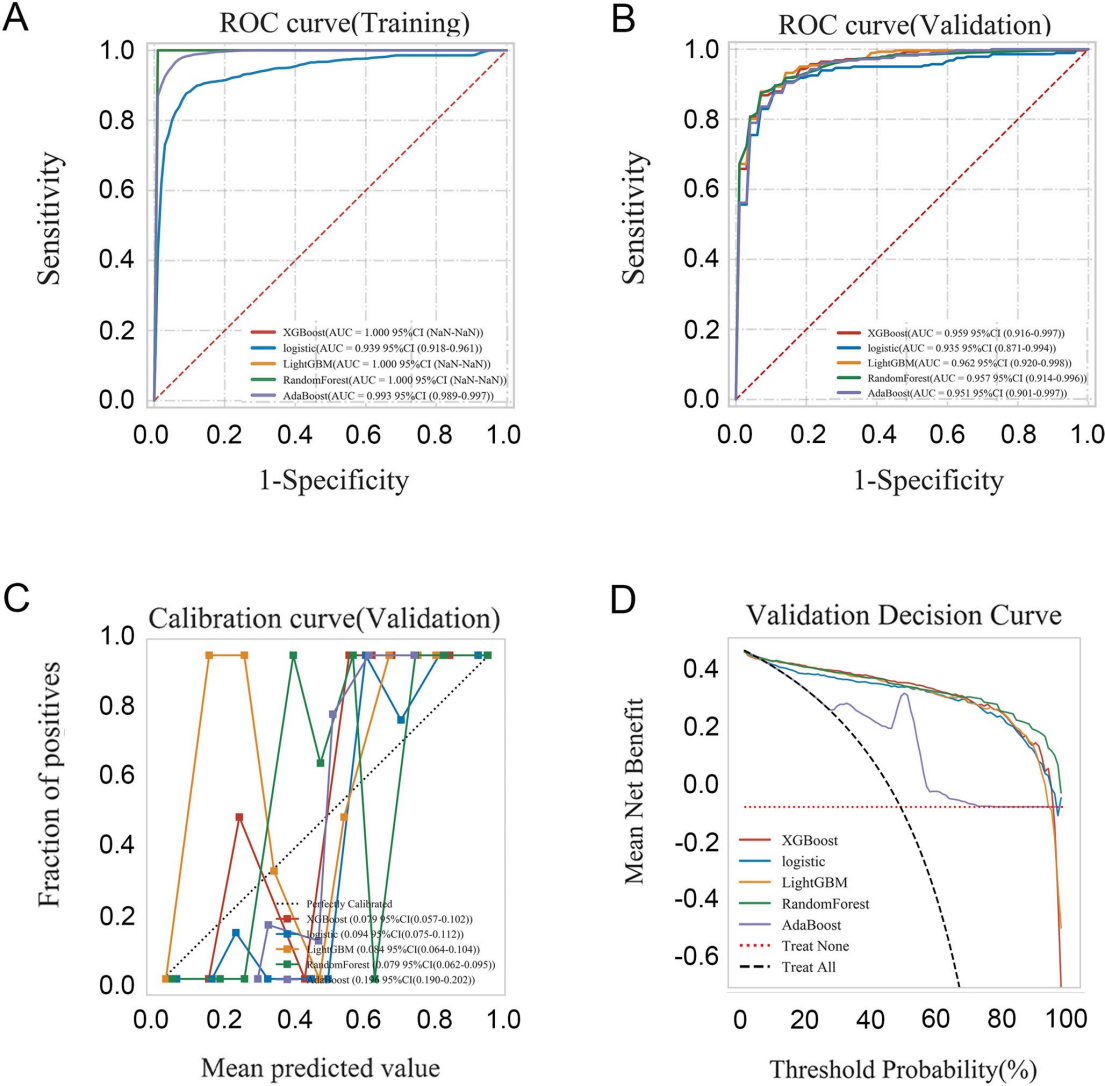
The comparison results of the five models for differentiation between thalassemia and iron deficiency anemia: Extreme Gradient Boosting (XGBoost), Logistic Regression (LR), Light Gradient Boosting Machine (LightGBM), Random Forest (RF) and Adaptive Boosting (AdaBoost). (**A**–**B**) ROC curve of the five models for training and validation test. (**C**–**D**) Calibration curve and validation curve for the five models. AUC, area under the curve; ROC, receiver operating characteristic

Five machine learning models—XGBoost, Logistic Regression (LR), LightGBM, Random Forest (RF), and AdaBoost—were trained and evaluated. All models exhibited strong discriminative performance, with area under the curve (AUC) values exceeding 0.93. LightGBM achieved the highest performance with an AUC of 0.962 (95% CI: 0.920–0.998) in the internal validation (Fig. 1; Table 2). Pairwise comparisons of model performance were conducted using DeLong’s tests. Although LightGBM consistently achieved higher AUC values than the other models, the differences were not statistically significant (Tables S1 and S2). Model robustness and clinical utility were further assessed using calibration plots and decision curve analysis.

**Table 2:** Result of the different machine learning models

Model type	AUC (95% CI)	Accuracy (95% CI)	Sensitivity (95% CI)	Specificity (95% CI)	PPV (95% CI)	NPV (95% CI)	F1 score (95% CI)
XGBoost	0.959(0.916-0.997)	0.889(0.870-0.908)	0.829(0.785-0.874)	0.946(0.915-0.977)	0.940(0.908-0.973)	0.857(0.823-0.890)	0.878(0.856-0.901)
logistic	0.935 (0.871-0.994)	0.884(0.860-0.907)	0.879(0.831-0.927)	0.888(0.848-0.929)	0.886(0.855-0.918)	0.891(0.851-0.931)	0.880(0.854-0.905)
LightGBM	0.962 (0.920-0.998)	0.880(0.858-0.902)	0.801(0.747-0.855)	0.956(0.930-0.982)	0.950(0.922-0.977)	0.839(0.801-0.877)	0.865(0.837-0.893)
RandomForest	0.957 (0.914-0.996)	0.901(0.873-0.929)	0.897(0.845-0.948)	0.905(0.857-0.953)	0.906(0.868-0.944)	0.907(0.868-0.946)	0.898(0.868-0.927)
AdaBoost	0.951 (0.901-0.997)	0.864(0.840-0.889)	0.883(0.831-0.934)	0.847(0.809-0.885)	0.850(0.821-0.879)	0.889(0.846-0.931)	0.863(0.837-0.890)

AUC, Area under the curve; PPV, positive predictive value; NPV, negative predictive value.

### Performance of the LightGBM model

**Fig. 2:**
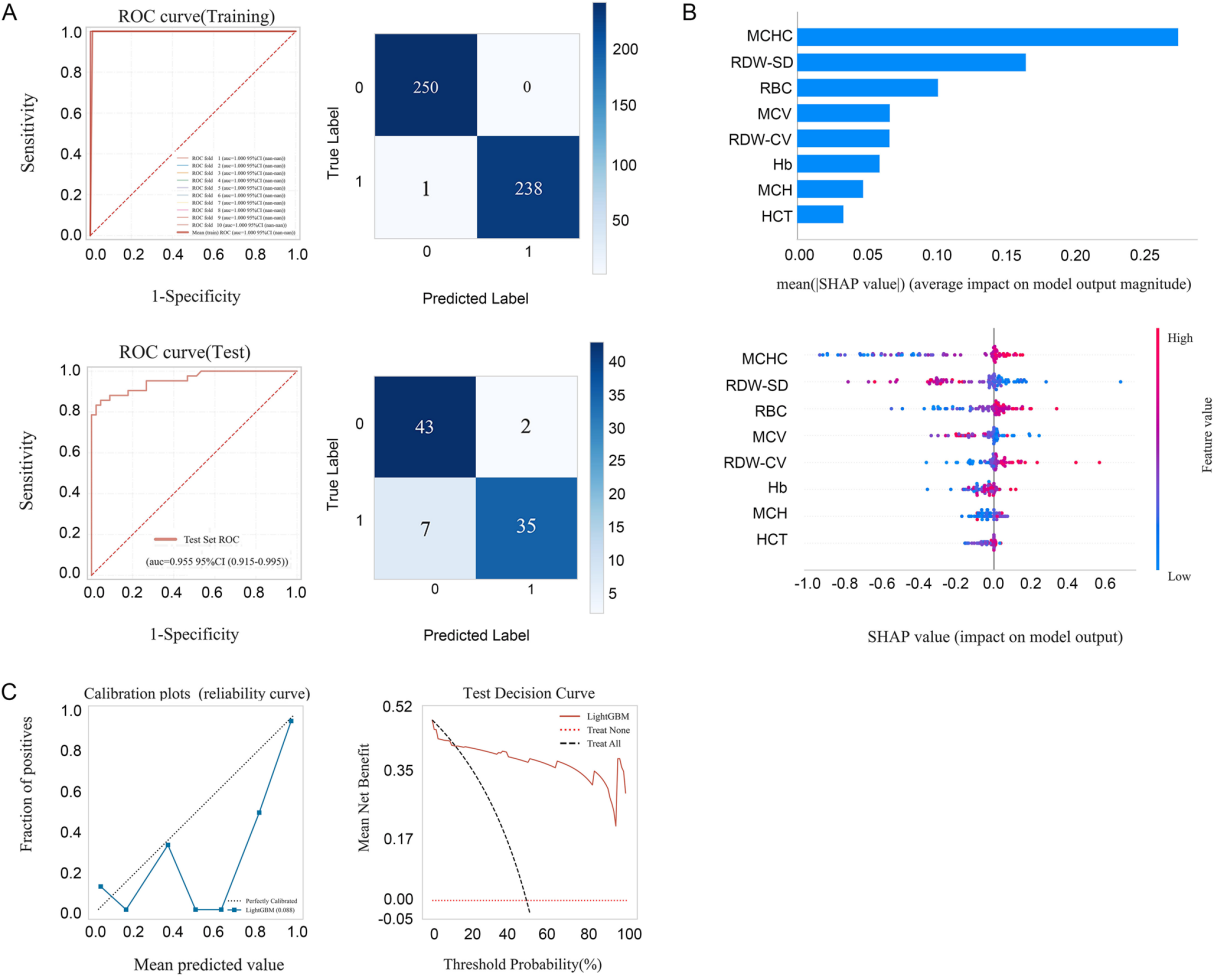
In the internal testing set, the LightGBM model for differentiation between thalassemia and iron deficiency anemia built by seven key features (MCHC, MCV, RDW-SD, RDW-CV, RBC, Hb, and HCT). (A) ROC curve for the training and testing set. (B) The SHAP values. Each feature’s SHAP value indicates its contribution to the model’s prediction. (C) The calibration plots and test decision curve of the XGBoost model. AUC, area under the curve; LightGBM, Light Gradient Boosting Machine; ROC, receiver operating characteristic; SHAP, SHapley Additive exPlanations; MCHC, mean corpuscular hemoglobin concentration; MCV, mean corpuscular volume; RDW-SD, red cell distribution width–standard deviation; RDW-CV, red cell distribution width–coefficient of variation; RBC, red blood cell count; Hb, Hemoglobin; HCT, hematocrit

As shown in Table 3; Fig. 2, the LightGBM model exhibited excellent diagnostic performance, with an AUC of 0.953 (95% CI: 0.938–0.968), accuracy of 86.9%, sensitivity of 79.9%, specificity of 93.6%, and F1-score of 0.856. Notably, the model achieved a Youden’s index of 0.857 and a diagnostic odds ratio (DOR) of 329.12 (95% CI: 167.422–490.808), reflecting strong discriminative ability and clinical applicability. Positive and negative likelihood ratios (PLR and NLR) further supported the model’s reliability, with PLR of 17.776 and NLR of 0.077. SHAP (SHapley Additive exPlanations) analysis revealed that MCHC and RDW-SD were the most influential predictors, followed by RBC, MCV, RDW-CV, Hb, MCH, and HCT (Fig. 2B).

**Table 3:** Performance of the built machine learning model

	Mean	95% CI Lower	95% CI Upper
AUC	0.953	0.938	0.968
Cutoff	0.841	0.824	0.858
Accuracy	0.869	0.837	0.901
Sensibility	0.799	0.753	0.846
Specificity	0.936	0.899	0.973
PPV	0.926	0.884	0.968
NPV	0.832	0.796	0.868
F1 score	0.856	0.821	0.891
Kappa	0.738	0.673	0.802
Youden's index	0.857	0.806	0.909
Positive LR	17.776	11.124	24.428
Negative LR	0.077	0.046	0.107
DOR	329.12	167.422	490.808

AUC,  area under the curve; PPV, positive predictive value; NPV, negative predictive value; Positive LR, positive and negative likelihood ratios; Negative LR, negative likelihood ratios; DOR, diagnostic odds ratios.

**Fig. 3:**
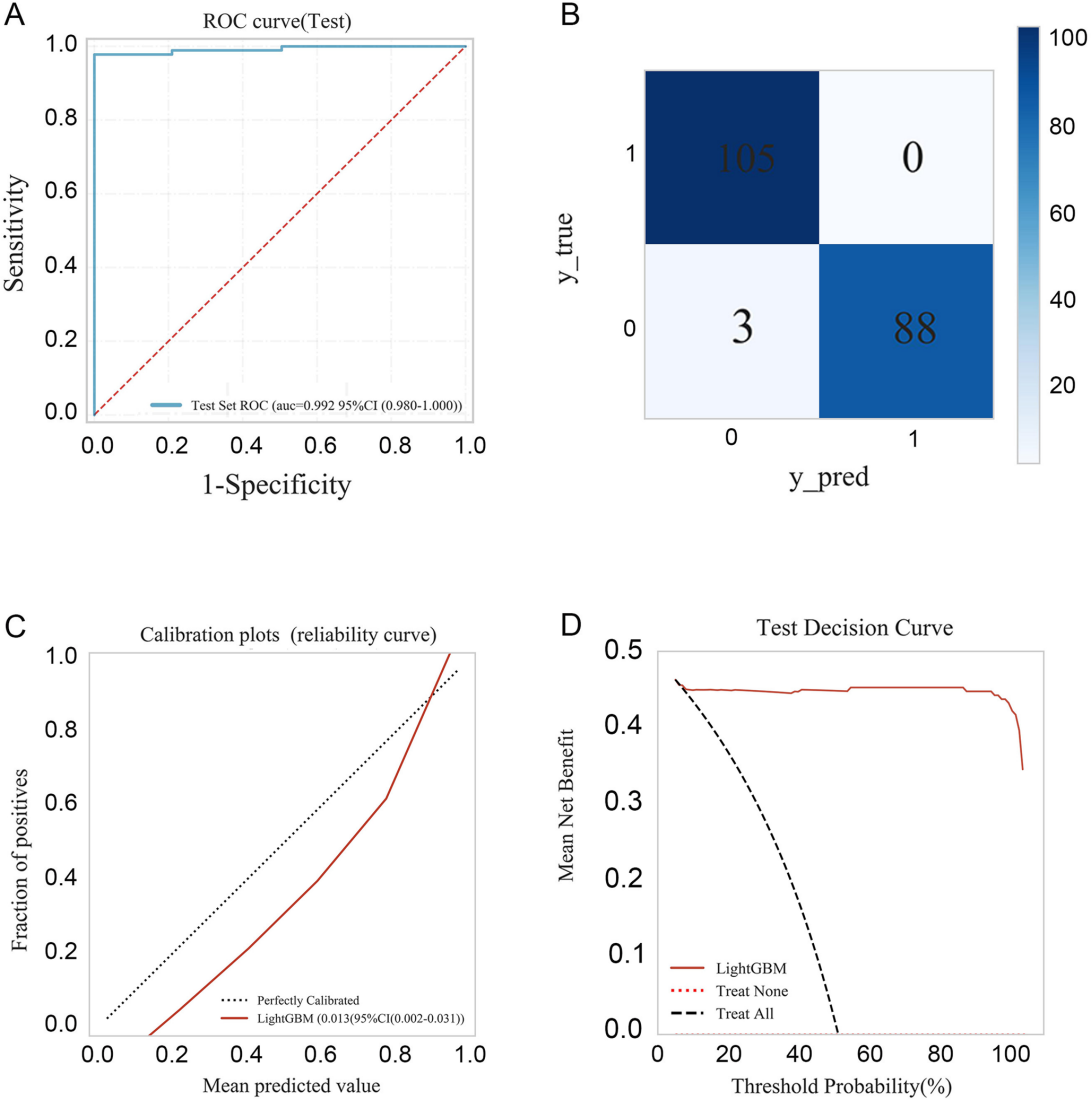
The results of the external validation of the model. (**A**–**B**) ROC curve and comprehensive confusion matrix of the external test. (**C**–**D**) The calibration plots and test decision curve of the external test. AUC, area under the curve; ROC, receiver operating characteristic

Importantly, the model demonstrated excellent generalizability in the external validation cohort, achieving an AUC of 0.992, accuracy of 98.5%, sensitivity of 96.7%. Positive predictive value (PPV), negative predictive value (NPV), and F1 scores were consistently high across all datasets (Table 4). The ROC curve and confusion matrix indicated high classification accuracy, with 105 IDA and 88 TT patients correctly identified and only three misclassified cases in total (Fig. 3A-B). The calibration plot revealed close agreement between predicted probabilities and observed outcomes, with a Brier score of 0.013 (95% CI: 0.002–0.031), indicating good reliability of probability estimation (Fig. 3C). Furthermore, decision curve analysis (DCA) demonstrated that the LightGBM model provided superior net clinical benefit across a wide range of threshold probabilities (Fig. 3D).

**Table 4:** Results of external validation of the machine learning model

	AUC	Accuracy	Sensibility	Specificity	PPV	NPV	F1 score
External validation	0.992	0.985	0.967	1.000	1.000	0.972	0.983

*AUC* area under the curve, *PPV* positive predictive value, *NPV* negative predictive value

The LightGBM model demonstrated superior diagnostic performance compared with most conventional discrimination formulas (Table S3). Notably, when compared with Index26, which was the best-performing traditional index, LightGBM achieved higher accuracy (86.9% vs. 84.67%) and a markedly elevated diagnostic odds ratio (DOR: 329.12 vs. 48.87), underscoring its enhanced discriminative power.

### Model interpretability and application for clinical utility

To enhance model interpretability, we employed SHAP (SHapley Additive exPlanations) values, an open-source methodology that provides both computational and graphical elucidation of feature contributions. As intuitively illustrated in Fig. 2, MCHC and RDW-SD were identified as the most significant features for distinguishing IDA from TT. The final prediction model was deployed as a freely accessible online calculator to support clinical utility. Users can input the seven key laboratory parameters to obtain a probabilistic prediction of the diagnosis. The web application is available at:

https://www.xsmartanalysis.com/model/list/predict/model/html?mid=31316&symbol=5eR1769cC6515Qv2hd32.

## Discussion

Iron deficiency anemia (IDA) and thalassemia trait (TT) represent the most prevalent causes of microcytic hypochromic anemia worldwide. Accurate differentiation between IDA and TT remains a diagnostic challenge due to their overlapping hematological profiles. This distinction is clinically crucial: inappropriate iron supplementation in TT carriers risks iron overload and long-term organ damage, whereas failure to detect carriers compromises timely genetic counseling and preconception screening.

In our comparative analysis, we benchmarked the performance of our LightGBM-based machine learning model against previously published hematological discrimination formulas, including Telmissani-MCHD, Huber-Herklotz, Kerman I/II, Sirdah, Ehsani, Nishad, and Index26 [17]. These indices, while widely used in resource-limited settings, often exhibit variable accuracy due to population heterogeneity and limited generalizability [10, 18]. However, their diagnostic accuracy is highly influenced by demographic factors including age, sex, and ethnic background, leading to inconsistent sensitivity and specificity across populations [19, 20]. By contrast, the LightGBM model demonstrated superior diagnostic accuracy and a markedly higher diagnostic odds ratio compared with most conventional indices. Notably, even when benchmarked against Index26—the best-performing traditional formula in our dataset—our model still outperformed it in both accuracy and DOR. In our study, seven key hematological parameters were identified as the most informative features for differentiating IDA from TT, with MCHC and RDW-SD contributing the greatest discriminative power. We applied several machine learning algorithms, all of which showed strong predictive capability. Among them, the LightGBM-based model that we developed demonstrated superior accuracy and generalizability, highlighting its robustness and potential clinical utility as a reliable tool for distinguishing IDA from TT.

LightGBM is a highly efficient gradient boosting framework that utilizes a leaf-wise tree growth strategy, optimizing split points and accelerating training through feature grouping and down-sampling. This approach enhances predictive accuracy while mitigating overfitting via regularization [21]. Compared to other machine learning methods reported in the literature—such as random forest (RF), support vector machines (SVM), and conventional gradient boosting (GB)—LightGBM offers superior scalability, interpretability, and computational efficiency. For instance, while RF and GB models have shown accuracies above 90% and AUCs up to 0.996 in training, their performance often declines in external testing for differentiating between IDA and TT [14]. Similarly, SVM models, though capable of handling imbalanced data, function as “black boxes” with limited interpretability [22, 23]. LightGBM balances high performance with practical transparency, making it particularly suitable for clinical decision support.

Consistent with prior studies, MCHC emerged as the most influential feature in our model. MCHC reflects the hemoglobin concentration within individual red blood cells and is markedly reduced in IDA due to impaired hemoglobin synthesis secondary to iron deficiency. In contrast, thalassemia trait exhibits relatively preserved MCHC, as iron metabolism remains intact despite globin chain imbalances. This pathophysiological distinction underpins the strong discriminative power of MCHC. Our findings align with those of Tepakhan et al., who also identified MCHC as a top predictors in RF and GB models [14]. Other studies have similarly highlighted MCV, MCH, RDW, and Hb as critical variables [12, 13]. To facilitate clinical translation, we developed a user-friendly, web-based prediction tool utilizing the LightGBM framework. This platform enables clinicians to input routine CBC parameters and receive immediate probability scores, guiding further diagnostic testing in patients with unexplained microcytic anemia. The tool is publicly accessible at:https://www.xsmartanalysis.com/model/list/predict/model/html?mid=27642&symbol=81bL7ky56979ku46mP71. By streamlining the diagnostic pathway, this tool may reduce redundant laboratory testing, minimize patient blood draw requirements, and lower overall healthcare expenditures.

### Limitations

This study has several limitations. First, the model was built using a limited set of laboratory parameters. Expanding the feature space to include additional clinical and genetic variables could improve discriminative performance. Second, although the sample size was sufficient for model development, larger multi-center datasets encompassing diverse ethnic and age groups would enhance generalizability. Finally, while the model demonstrated robust performance in external validation from local centers, further evaluation in international and multi-ethnic cohorts is necessary to fully assess its robustness and clinical applicability across various healthcare settings.

## Conclusion

In conclusion, our findings demonstrate that the LightGBM-based machine learning model outperforms both conventional discriminant formulas and other machine learning algorithms in distinguishing IDA from TT. The proposed approach leverages an advanced gradient-boosting framework to achieve superior predictive accuracy, robustness, and generalizability compared with existing methods. While traditional statistical indices and earlier machine learning models have provided valuable diagnostic support, our results indicate that modern ensemble learning techniques like LightGBM offer enhanced capability in capturing complex hematological patterns associated with these conditions.

Accurate differentiation between these disorders carries significant clinical implications, including appropriate treatment selection, avoidance of iron overload in individuals with TT, and informed genetic counseling—particularly in the context of family planning and prenatal screening. Furthermore, the implementation of such a model in clinical practice may streamline diagnostic pathways, reduce reliance on costly and time-consuming confirmatory tests, and facilitate earlier intervention. Future studies should focus on external validation in diverse populations and integration of additional clinical and genetic features to further enhance model performance and applicability.

## Supplementary Information

Below is the link to the electronic supplementary material.

Supplementary Material 1 (46.3 KB)

Supplementary Material 2 (12.1 KB)

Supplementary Material 3 (12.6 KB)

Supplementary Material 4 (24.3 KB)

## Data Availability

All data supporting the findings of this study are available within the paper and its Supplementary Information.
