# Supplementary material for: Comparison of the diagnostic performance of machine learning algorithms for differentiating iron deficiency anemia and thalassemia
Source: Ann Hematol. 2026 Mar 4;105(4):159. doi: 10.1007/s00277-026-06894-5 (PMC12960304; doi:10.1007/s00277-026-06894-5)
Supplement: Supplementary file 1 — Supplementary Material 1 (46.3 KB) [file 277_2026_6894_MOESM1_ESM.zip › Table S2.docx]

| Table S2: P-values from DeLong’s test for pairwise comparisons of AUCs between different machine learning models. | | | | | |
| --- | --- | --- | --- | --- | --- |
|  | XGB Classifier | Logistic Regression | LGBM Classifier | Random Forest Classifier | AdaBoost Classifier |
| XGB Classifier | / | 0.422 | 0.392 | 0.437 | 0.516 |
| Logistic Regression | 0.422 | / | 0.327 | 0.286 | 0.421 |
| LGBM Classifier | 0.392 | 0.327 | / | 0.511 | 0.550 |
| Random Forest Classifier | 0.437 | 0.286 | 0.511 | / | 0.482 |
| AdaBoost Classifier | 0.516 | 0.421 | 0.550 | 0.482 | / |
| AUC, area under the curve. | | | | | |
