# Supplementary material for: Comparison of the diagnostic performance of machine learning algorithms for differentiating iron deficiency anemia and thalassemia
Source: Ann Hematol. 2026 Mar 4;105(4):159. doi: 10.1007/s00277-026-06894-5 (PMC12960304; doi:10.1007/s00277-026-06894-5)
Supplement: Supplementary file 1 — Supplementary Material 1 (46.3 KB) [file 277_2026_6894_MOESM1_ESM.zip › Table S3.docx]

| Table S3: Comparison of Diagnostic Performance Between Traditional Discriminant Formulas and the Proposed Machine Learning Model | | | | | |
| --- | --- | --- | --- | --- | --- |
| Discriminant Formula | Youden’s Index (%) | Accuracy (%) | LR + (%) | LR − (%) | DOR (%) |
| England and Fraser (E&F) | 48.35 | 72.11 | 4.31 | 0.43 | 10.02 |
|  | (40.09–55.88) | (69.06–75) | (3.34–5.56) | (0.39–0.49) | (7.092–13.93) |
| RBC | 48.35 | 76.59 | 2.27 | 0.22 | 10.32 |
|  | (39.92–56.17) | (73.68–79.32) | (1.98–2.60) | (0.17–0.28) | (7.47–14.33) |
| Mentzer | 67.66 | 84.78 | 4.17 | 0.14 | 29.79 |
|  | (60.17–74.25) | (82.28–87.06) | (3.42–5.08) | (0.11–0.18) | (20.67–43.09) |
| Srivastava | 55.67 | 77.29 | 3.9 | 0.31 | 12.58 |
|  | (47.39–63.17) | (74.42–79.98) | (3.15–4.84) | (0.27–0.36) | (9.07–17.34) |
| Shine and Lal (S&L) | 17.57 | 66.37 | 1.21 | 0 | ∞ |
|  | (12.80–21.83) | (63.19–69.44) | (1.16–1.27) |  |  |
| Bessman | –13.67 | 36.38 | 0.32 | 1.17 | 0.27 |
|  | (–20.02–7.31) | (33.25–39.61) | (0.22–0.46) | (1.11–1.24) | (0.18–0.42) |
| Ricerca | 5.72 | 61.3 | 1.06 | 0.19 | 5.58 |
|  | (1.97–9.60) | (58.04–64.48) | (1.03–1.09) | (0.08–0.42) | (2.46–13.33) |
| Green and King (G&K) | 65.24 | 83.35 | 4.06 | 0.17 | 23.88 |
|  | (57.53–72.08) | (80.76–85.72) | (3.33–4.95) | (0.14–0.21) | (16.74–33.80) |
| Das Gupta | 31.56 | 71.22 | 1.49 | 0.13 | 11.46 |
|  | (24.52–38.31) | (68.16–74.15) | (1.38–1.62) | (0.09–0.19) | (7.38–18.31) |
| Jayabose (RDWI) | 56.87 | 81.04 | 2.59 | 0.12 | 21.58 |
|  | (49.20–63.83) | (78.33–83.54) | (2.26–2.98) | (0.09–0.16) | (15.23–32.96) |
| Telmissani – MCHD | 1.83 | 59.65 | 1.02 | 0.48 | 2.13 |
|  | (–1.27–5.16) | (56.37–62.86) | (1.00–1.04) | (0.21–1.10) | (0.90–5.05) |
| Telmissani – MDHL | 42.1 | 68.36 | 3.94 | 0.51 | 7.73 |
|  | (33.80–49.75) | (65.22–71.37) | (3.04–5.11) | (0.46–0.56) | (5.53–10.85) |
| Huber – Herklotz | 8.48 | 48.4 | 1.6 | 0.9 | 1.78 |
|  | (1.05–15.63) | (45.10–51.71) | (1.19–2.16) | (0.85–0.96) | (1.25–2.54) |
| Kerman I | 56.3 | 81.15 | 2.48 | 0.09 | 27.56 |
|  | (48.85–63.06) | (78.45–83.64) | (2.17–2.83) | (0.06–0.13) | (17.97–41.94) |
| Kerman II | 70.8 | 86 | 4.97 | 0.14 | 35.5 |
|  | (63.52–77.13) | (83.57–88.19) | (3.98–6.20) | (0.11–0.18) | (24.66–52.38) |
| Sirdah | 68.91 | 83.68 | 7.07 | 0.22 | 32.14 |
|  | (61.61–75.24) | (81.11–86.03) | (5.30–9.43) | (0.19–0.27) | (21.60–46.67) |
| Ehsani | 70.36 | 85.89 | 4.77 | 0.14 | 34.07 |
|  | (63.06–76.72) | (83.45–88.09) | (3.85–5.92) | (0.11–0.17) | (24.26–51.49) |
| Keikhaei | 61.34 | 82.14 | 3.25 | 0.16 | 20.31 |
|  | (53.51–68.38) | (79.49–84.58) | (2.74–3.85) | (0.12–0.20) | (14.63–29.53) |
| Nishad | 62.32 | 81.92 | 3.71 | 0.19 | 19.53 |
|  | (54.40–69.39) | (79.26–84.37) | (3.07–4.49) | (0.15–0.24) | (13.83–27.31) |
| Wongprachum | 57.36 | 80.38 | 2.88 | 0.17 | 16.94 |
|  | (49.32–64.65) | (77.64–82.91) | (2.46–3.37) | (0.14–0.22) | (11.75–23.22) |
| Sehgal | 60.68 | 83.24 | 2.71 | 0.06 | 45.17 |
|  | (53.57–67.03) | (80.65–85.62) | (2.36–3.12) | (0.04–0.09) | (27.59–72.85) |
| Pornprasert | –43.57 | 26.79 | 0.32 | 2.21 | 0.15 |
|  | (–51.80 – –34.78) | (23.93–29.80) | (0.27–0.38) | (1.92–2.55) | (0.11–0.20) |
| Sirachainan | 10.8 | 51.82 | 1.43 | 0.86 | 1.66 |
|  | (2–19.37) | (48.51–55.12) | (1.16–1.76) | (0.78–0.93) | (1.25–2.24) |
| Bordbar | 52.61 | 80.15 | 2.18 | 0.05 | 43.6 |
|  | (45.61–58.97) | (77.41–82.70) | (1.94–2.44) | (0.03–0.08) | (24.88–75.14) |
| Matos and Carvalho | 58.04 | 78.94 | 3.83 | 0.27 | 14.2 |
|  | (49.85–65.44) | (76.14–81.55) | (3.12–4.70) | (0.23–0.32) | (10.25–19.66) |
| Janel (11T) | 68.5 | 83.24 | 7.67 | 0.24 | 31.96 |
|  | (61.24–74.79) | (80.65–85.62) | (5.66– | (0.20–0.28) | (21.86–48.09) |
| CRUISE | 49.34 | 75.08 | 2.79 | 0.32 | 8.72 |
|  | (40.69–57.33) | (72.13–77.87) | (2.35–3.31) | (0.27–0.38) | (6.46–11.86) |
| Index26 | 71.93 | 84.67 | 11.24 | 0.23 | 48.87 |
|  | (65.13–77.69) | (82.16–86.96) | (7.74–16.32) | (0.19–0.27) | (31.67–77.81) |
| LightGBM ML | 85.7 | 86.9 | 17.776 | 0.08 | 329.12 |
|  | (80.60-90.9) | (83.70-90.10) | (11.12-24.43) | (0.05-0.11) | (167.42-490.81) |
| LR, likelihood ratio; DOR, diagnostic odds ratio ; ML, machine learning. | | | | | |
