# Supplementary material for: Comparison of the diagnostic performance of machine learning algorithms for differentiating iron deficiency anemia and thalassemia
Source: Ann Hematol. 2026 Mar 4;105(4):159. doi: 10.1007/s00277-026-06894-5 (PMC12960304; doi:10.1007/s00277-026-06894-5)
Supplement: Supplementary file 1 — Supplementary Material 1 (46.3 KB) [file 277_2026_6894_MOESM1_ESM.zip › Table S1.docx]

| Table S1: Z-values from DeLong’s test for pairwise comparisons of AUCs between different machine learning models. | | | | | |
| --- | --- | --- | --- | --- | --- |
|  | XGBClassifier | LogisticRegression | LGBMClassifier | RandomForestClassifier | AdaBoostClassifier |
| XGBClassifier | / | 0.909 | 0.929 | 0.821 | 0.807 |
| LogisticRegression | 0.909 | / | 1.050 | 1.114 | 0.864 |
| LGBMClassifier | 0.929 | 1.050 | / | 0.716 | 0.715 |
| RandomForestClassifier | 0.821 | 1.114 | 0.716 | / | 0.802 |
| AdaBoostClassifier | 0.807 | 0.864 | 0.715 | 0.802 | / |
| AUC, area under the ROC curve. | | | | | |
